# Increased Expression of Bcl11b Leads to Chemoresistance Accompanied by G1 Accumulation

**DOI:** 10.1371/journal.pone.0012532

**Published:** 2010-09-02

**Authors:** Piotr Grabarczyk, Viola Nähse, Martin Delin, Grzegorz Przybylski, Maren Depke, Petra Hildebrandt, Uwe Völker, Christian A. Schmidt

**Affiliations:** 1 Molecular Hematology, Department of Hematology and Oncology, University Greifswald, Greifswald, Germany; 2 Department of Functional Genomics, Interfaculty Institute for Genetics and Functional Genomics, Greifswald, Germany; 3 Institute of Human Genetics, Polish Academy of Sciences, Poznan, Poland; St Georges University of London, United Kingdom

## Abstract

**Background:**

The expression of *BCL11B* was reported in T-cells, neurons and keratinocytes. Aberrations of *BCL11B* locus leading to abnormal gene transcription were identified in human hematological disorders and corresponding animal models. Recently, the elevated levels of Bcl11b protein have been described in a subset of squameous cell carcinoma cases. Despite the rapidly accumulating knowledge concerning Bcl11b biology, the contribution of this protein to normal or transformed cell homeostasis remains open.

**Methodology/Principal Findings:**

Here, by employing an overexpression strategy we revealed formerly unidentified features of Bcl11b. Two different T-cell lines were forced to express *BCL11B* at levels similar to those observed in primary T-cell leukemias. This resulted in markedly increased resistance to radiomimetic drugs while no influence on death-receptor apoptotic pathway was observed. Apoptosis resistance triggered by *BCL11B* overexpression was accompanied by a cell cycle delay caused by accumulation of cells at G1. This cell cycle restriction was associated with upregulation of *CDKN1C* (p57) and *CDKN2C* (p18) cyclin dependent kinase inhibitors. Moreover, p27 and p130 proteins accumulated and the *SKP2* gene encoding a protein of the ubiquitin-binding complex responsible for their degradation was repressed. Furthermore, the expression of the *MYCN* oncogene was silenced which resulted in significant depletion of the protein in cells expressing high *BCL11B* levels. Both cell cycle restriction and resistance to DNA-damage-induced apoptosis coincided and required the histone deacetylase binding N-terminal domain of Bcl11b. The sensitivity to genotoxic stress could be restored by the histone deacetylase inhibitor trichostatine A.

**Conclusions:**

The data presented here suggest a potential role of *BCL11B* in tumor survival and encourage developing Bcl11b-inhibitory approaches as a potential tool to specifically target chemoresistant tumor cells.

## Introduction

The *BCL11B* gene encodes a protein which was originally described as chicken ovalbumin upstream promoter transcription factor (COUP-TF)-interacting protein 2 (CTIP2) [1] and radiation induced tumor suppressor gene 1 (*RIT1*) [2]. Bcl11b belongs to C_2_H_2_-zinc finger Krueppel-like proteins, the largest family of transcription factors in eukaryotes. DNA binding is mediated *via* amino acids on the surface of an alpha-helix [3], [4]. Apart from the DNA binding region, Bcl11b possesses domains responsible for transcriptional regulation. The catalogue of proteins and protein complexes known to interact with Bcl11b has grown recently. It includes COUP-TF [5], the nucleosome re-modeling and histone deacetylation complex (NuRD) [6] and the ubiquitous transcription factor Sp1 [7]. Furthermore, recruitment of histone deacetylases (HDAC1 and HDAC2, resp. SIRT1) [6], [8] and the histone methyltransferase SUV39H1 by Bcl11b induces heterochromatin formation and makes it a potent transcriptional repressor [9]. Conversely, Bcl11b interaction with p300 co-activator on the upstream site 1 (US1) of the *IL-2* promoter results in transcriptional activation of *IL-2* expression in activated T-cells [10]. Interestingly, although interaction partners and their binding sequence have been revealed only a few direct target genes of *BCL11B* have been discovered to date. The *P57/KIP2* gene, i.e., a cyclin-dependent kinase inhibitor, is suppressed by Bcl11b [11]. In addition to *P57* and *IL-2* genes, the cancer Osaka thyroid oncogene (Cot) has been recently identified as a direct transcriptional target of Bcl11b. Similar to *IL-2*, the engagement of Bcl11b on the Cot promoter region led to induction of Cot expression and its kinase activity. This caused an augmented phosphorylation of IkappaB kinase which resulted in increased translocation of NF-kappaB to the nucleus and activation of its target genes [12]. The number of Bcl11b-regulated genes was recently extended to include the major cell cycle regulator and tumor suppressor *CDKN1A/p21WAF1* which was demonstrated to be repressed by Bcl11b acting *via* recruiting histone deacetylases and methyltransferases to the *p21WAF1* promoter [13].

The list of biological processes requiring *BCL11B* is constantly expanding. It includes the regulation of T-cell differentiation [14], normal development of central nervous system (CNS) during embryogenesis [15], [16] and the maintenance of the latent state of human immunodeficiency virus (HIV) infections [9]. Of note, *BCL11B* which has initially been thought to be of importance to the immune and central nervous systems seems to have a considerably broader impact. The results published within the last years showed the requirement for *BCL11B* in developing skin [17], where it regulates keratinocyte proliferation and the late differentiation phases determining the process of skin morphogenesis [18]. Moreover, normal tooth development also required *BCL11B* expression and was significantly impaired in *BCL11B*-deficient mice which was accompanied by the decreased expression of ameloblast marker genes and transcription factors driving odontogenesis [19]. The rapidly growing relevance of *BCL11B* for the normal development of different organs and pathogenesis of various diseases requires further investigation of cellular and molecular mechanisms involving Bcl11b.

The recently acquired and already established data suggest a critical role of *BCL11B* in three major cellular processes: proliferation, survival and differentiation. The *BCL11B* knockout mouse model revealed the apoptotic phenotype of Bcl11b^−/−^ thymocytes accompanied by decreased expression of *BCLxL* and *BCL-2* genes [14]. The earlier finding that ectopic expression of *BCL11B* in HeLa cells caused cell cycle retardation inspired the authors to develop a hypothesis of unscheduled proliferation as a primary cause of cell death in Bcl11b-depleted cells. The suppressive influence of accumulated Bcl11b on cell cycle progression was later confirmed in a hematopoietic cell line [20]. However, the mechanism responsible for the reduced proliferation has not been elucidated to date. Moreover, the recently described Bcl11b-mediated transcriptional repression of *P57/KIP2* and *p21WAF1* cyclin-dependent kinase inhibitors responsible for cell cycle restriction should lead to effects opposite to the observed cell cycle retardation [13], [21].

Using a RNA interference approach, we could reproduce the apoptotic phenotype in transformed T cell lines but not in normal mature cells which suggested that apoptosis following Bcl11b depletion is transformation-dependent [22]. These data were confirmed by other reports showing not only reduced survival associated with *BCL11B* knockdown but also impaired response to DNA damage, disabled checkpoint activation and replication stress [23]. These two reports emphasize the anti-apoptotic role of Bcl11b but also uncover its potential function in maintaining genome stability, two features which might contribute to the malignant transformation.

The role of *BCL11B* in the pathogenesis of hematological diseases is still a matter of debate. In humans overexpression of *BCL11B* has been linked to lymphoproliferative disorders like the T-cell acute lymphoblastic leukemia (T-ALL) [24], [25] and an acute form of adult T-cell leukemia/lymphoma [26]. Furthermore, *BCL11B* induction correlated with the low differentiation status in head and neck squamous cell carcinoma where Bcl11b co-localized with the cancer stem cell marker BMI-1 [27]. In contrast, mouse models of T-cell leukemia revealed frequent homozygous deletions or mutations within the *BCL11B* locus [28]. Moreover, the loss of one allele was identified as a factor predisposing to lymphoma development in p53 (+/−) mice which implicated that *BCL11B* is a haploinsufficient tumor suppressor for thymoma progression in this genetic background and that deletion of one gene copy supports uncontrolled growth [29]. These inconsistencies between animal models and human diseases intensify the dispute on oncogenic or anti-neoplastic properties of *BCL11B*.

Here we show that *BCL11B* possesses features which, depending on the expression level and the cellular context, could predispose it to the role of both tumor suppressor and a gene supporting survival of transformed cells. Employing the gene overexpression strategy we demonstrated the relevance of Bcl11b in cell cycle control, chemoresistance and cell death. Our data provide further encouragement for the development of BCL11b-targeting, anti-neoplastic strategies. The attractiveness of such approaches increases together with the accumulating proofs for *BCL11B's* role in different malignant diseases.

## Results

### Elevated level of Bcl11b protects transformed T cells from DNA-damage-induced apoptosis but does not interfere with death-receptor pathway

**Figure 1:**
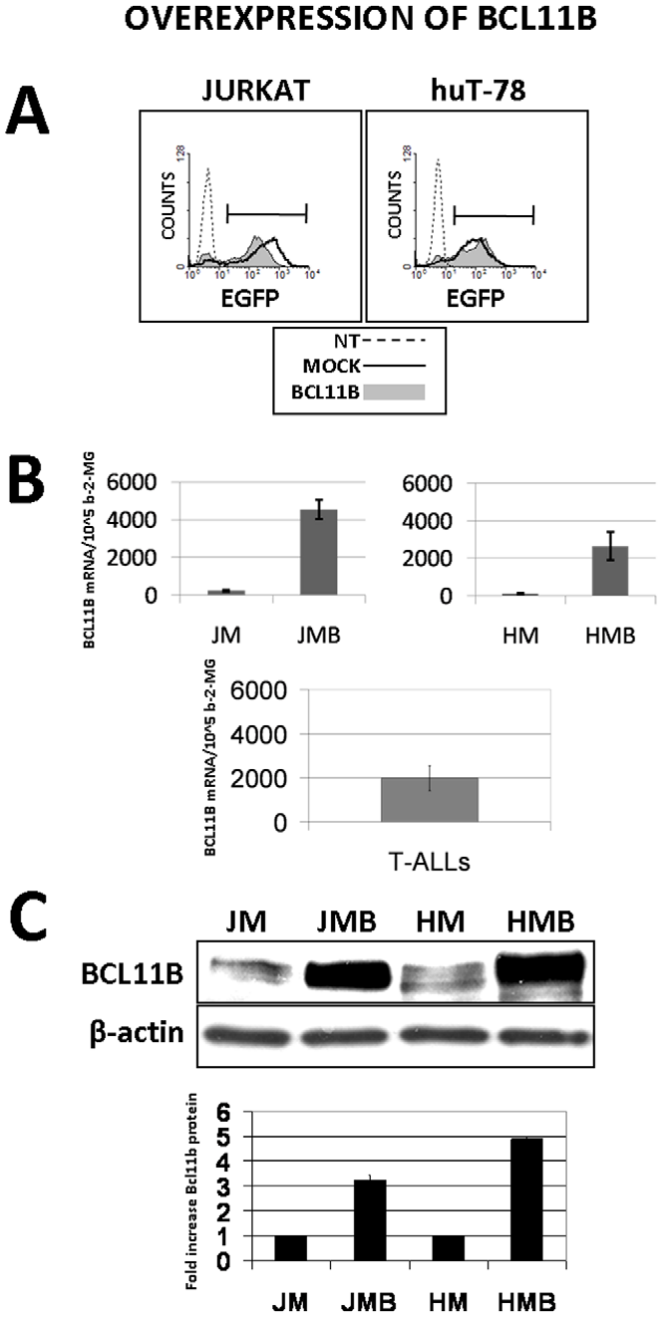
Evaluation of retrovirus-mediated *BCL11B* transfer into Jurkat and huT-78 cell lines. (**A**) The cells transduced with empty (mock) or *BCL11B*-encoding retroviral vector were analyzed by FACS 48h after transduction. The gene transfer efficiency was evaluated by measuring the percentage of GFP-positive cells. Non-treated (NT) parental cell lines served as background fluorescence control (**B**) The level of *BCL11B* mRNA was determined by semi-quantitative RT-PCR assay using β-2-microglobulin as a reference gene in mock-transduced (JM, HM) or *BCL11B*-transduced cell lines (JMB, HMB). For comparison *BCL11B* expression was measured in 20 primary T-cell leukemia cases (T-ALLs) (**C**) The overexpression of *BCL11B* was confirmed on protein level by immunodetection using the polyclonal anti-Bcl11b rabbit antibodies. The equal protein loading was confirmed by detecting β-actin.

The mechanisms of cell death caused by Bcl11b-depletion were already described, but the potential contribution of elevated Bcl11b to cell survival and proliferation has not been explored in detail yet. To address this question, we developed the retroviral-vector-based *BCL11B* overexpression system in two different T cell lines: Jurkat and huT78. The transduction efficiency monitored by measurements of green fluorescence encoded by the reporter gene GFP reached 85% (Fig. 1a). This resulted in an over 20-fold upregulation of *BCL11B* mRNA expression which reached (huT78) or exceeded (Jurkat) the average expression level measured in primary T-cell leukemia samples (Fig. 1b). On protein level a 3–5 fold increase of the Bcl11b-specific signal was observed (Fig. 1c). To ensure an equal and high transgene expression and the reproducibility of further experiments, mock and *BCL11B*-transduced cells were additionally sorted by FACS to eliminate non-modified cells.

**Figure 2:**
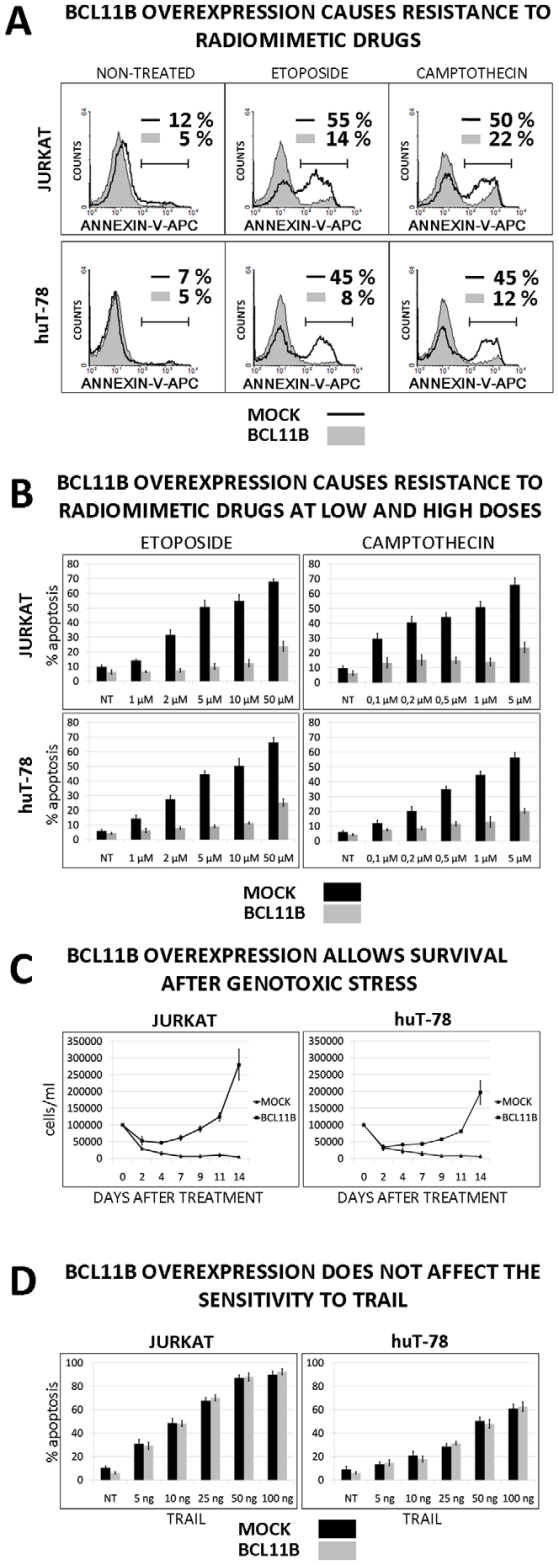
Influence of Bcl11b level on apoptosis and cell survival. (**A**) Mock- and *BCL11B*-transduced cells were treated or not with etoposide (10 µM) and camptothecin (2 µM). The influence of the treatment on cell viability was evaluated 6h later using a allophycocyanin-labeled Annexin-V binding assay and subsequent FACS analysis. The data represent one of at least three independent experiments. (**B**) The 6 hours etoposide and camptothecin treatments followed by the Annexin-V binding assay were performed using increasing concentrations of the radiomimetic drugs. (**C**) Mock- or *BCL11B*-transduced Jurkat and huT-78 cells were pulse-treated with 50 µM etoposide. The survival rate was determined by live cell counting performed every 48h after etoposide removal. (**D**) The cells with endogenous and elevated *BCL11B* expression were incubated with increasing amounts of the recombinant tumor necrosis factor related apoptosis inducing ligand (rTRAIL) for 6h. The procedure was followed by FACS-based Annexin-V binding detection. The data shown in **B**, **C** and **D** represent mean values ± standard deviations (SD) obtained from at least three independent experiments. Statistical significance was calculated using t-Student test. *p<0.01.

The elevated Bcl11b level itself did not cause significant difference in cell viability compared to mock-transduced cells as measured by Annexin-V binding assay. In contrast, induction of apoptosis with DNA-damaging agents such as etoposide (ETO) and camptothecin (CAM) was ineffective in Jurkat and huT78 cells expressing high levels of *BCL11B* compared to the parental cell lines (Fig. 2a). This finding was confirmed for a broad range of ETO and CAM concentrations (Fig. 2b) and for other radiomimetic agents like actinomycin D or dihydroethidium (not shown).

We next investigated whether forced expression of *BCL11B* changed only the kinetics of cell death or whether it could also influence the long-term survival upon genotoxic stress. The mock- and *BCL11B*-transduced cells were treated with etoposide for 6h after which the drug was removed by washing. The survival rate was assessed by counting live cells every 48h for 2 weeks. The 6h treatment disabled expansion of mock transduced cells while the cells with elevated Bcl11b restarted proliferation after 4–7 days (Fig. 2c). The same approach employing camptothecin and actinomycin D validated these findings (not shown). This observation supports the notion that Bcl11b not only altered the kinetics of apoptosis induced by DNA damage but also allowed the survival of cells temporarily exposed to genotoxic stress.

To acquire more insight into the Bcl11b-mediated apoptosis resistance, we exposed the cells to the death-receptor ligand TRAIL. Surprisingly, we did not observe any protection against TRAIL treatment in BCL11B-transduced cells neither at low nor at high doses (Fig. 2d). This indicated that Bcl11b provided selective resistance to genotoxic stress while it had no effect on apoptosis triggered by death-receptors.

### Bcl11b blocks the initiation phase of DNA-damage-induced cell death

**Figure 3:**
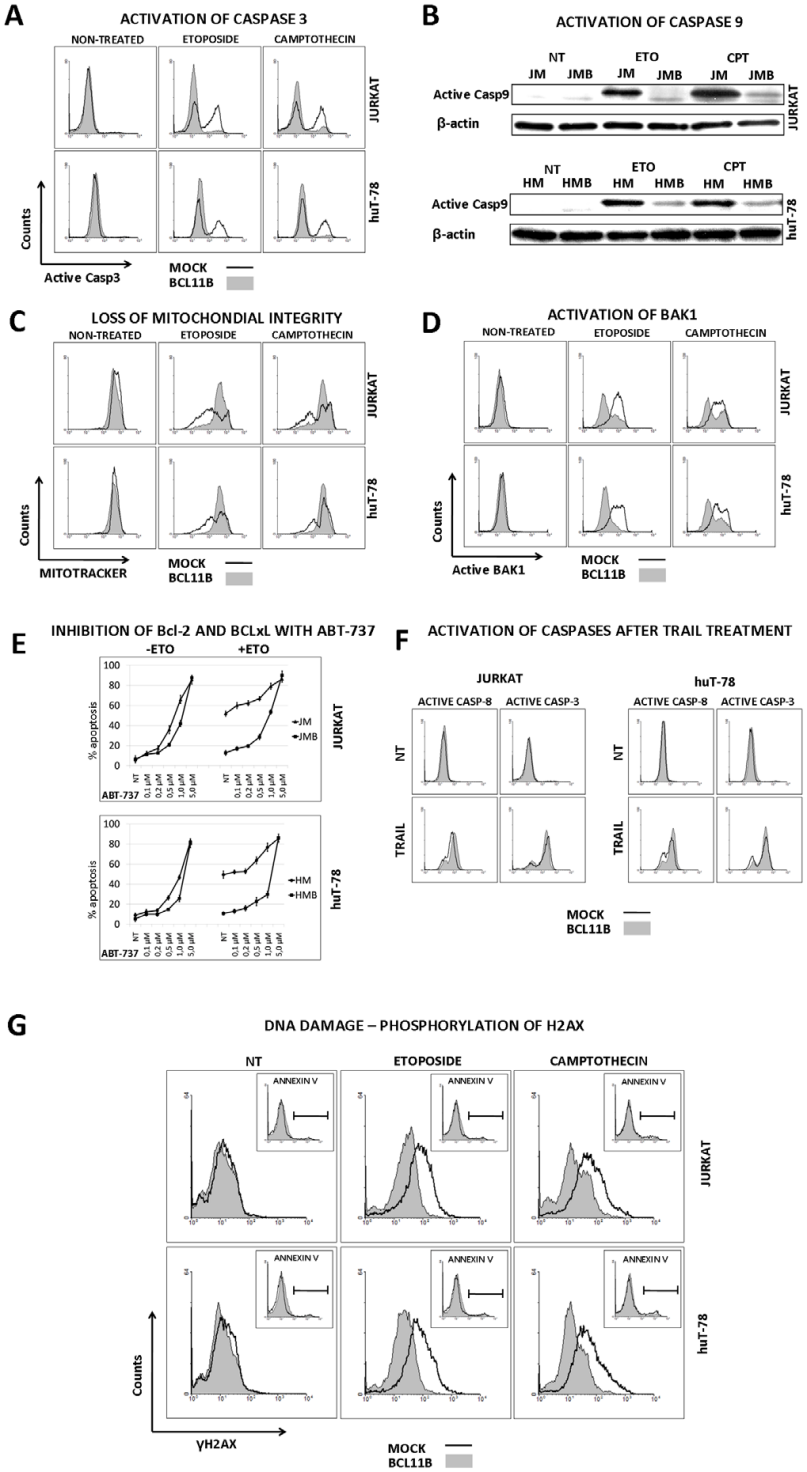
Investigation of DNA-damage- and TRAIL-induced apoptosis pathways. (**A**) Jurkat and huT-78 cells expressing normal and high *BCL11B* levels were incubated with ETO and CAM for 4h. The cleaved (active) form of Caspase 3 was stained using a fluorescently labeled antibody and measured by FACS in fixed/permeabilized cells. (**B**) Protein lysates obtained from the cells exposed to ETO and CAM for 4h were checked for the presence of activated Caspase 9 by immunodetection of its cleaved variant with Western Blotting. (**C**) A portion of mock- and *BCL11B*-modified cells, treated or not with ETO and CAM for 2h was incubated with the intact-mitochondria-accumulating dye (Mitotracker) for 1h. The accumulation of the dye reflecting the non-disrupted state of mitochondrial membranes was measured by FACS 3h after triggering DNA damage. (**D**) At the same time-point, a fraction of fixed and permeabilized cells was stained using the antibody recognizing the activated variant of Bak-1 protein. (**E**) Jurkat and huT-78 cells with endogenous and increased *BCL11B* expression were incubated with growing concentration of Bcl-2 and Bcl-xL inhibitor ABT-737 and treated or not with Eto (10 µM) or CAM (2 µM). 6h later the viability of the cells was tested using Annexin-V binding assay followed by FACS analysis. (**F**) The cell transduced with empty- or transgene-encoding vectors were incubated with 50 ng/ml rTRAIL, collected, fixed and permeabilized 4h after initiation of treatment. The activation of Caspase 8 and Caspase 3 was determined using cleaved variant-specific antibodies followed by FACS measurement. (**G**) The mock- and *BCL11B*-modified cells were treated with ETO and CAM and two hours after drug administration the cells were tested for viability using a Annexin V-binding assay (insets). The remaining cells were fixed/permeabilized and stained with a phospho-specific, AlexaFluor-647-labeled H2AX (γH2AX) antibody. The γH2AX-positive cells were detected by FACS analysis. Parts A, B, C, D, F and G show data representative of at least three independent experiments.

To explore possible mechanisms of enhanced survival in *BCL11B* overexpressing cells exposed to genotoxic stress we performed a retrograde analysis of the apoptosis cascade. After ETO or CAM treatment the activity of caspase 3 assessed by immunodetection with antibodies raised against the active/cleaved variant was markedly lower in cells transduced with the *BCL11B*-encoding vector (Fig. 3a). Also the apical caspase 9 acting on the intrinsic DNA-damage-induced apoptotic pathway showed reduced activity in the same conditions (Fig. 3b). Labeling of the cells with the live-mitochondria-specific dye Mitotracker DeepRed revealed a significant loss of mitochondrial integrity in mock-transduced cells while in *BCL11B*-overexpressing cells the pattern of staining remained unchanged after CAM treatment (Fig. 3c). Moreover, the activation of Bak-1, a pro-apoptotic member of the Bcl-2 protein family, which initiates the loss of mitochondrial outer membrane potential (MOMP) as a result of DNA damage, was evident in cell expressing endogenous levels of *BCL11B* but only minor in cells with elevated Bcl11b (Fig. 3d). Sensitization of cells to DNA-damage induced apoptosis with the Bcl-2/BCLxL inhibitor ABT-737 did not overcome the protective effect of Bcl11b at low to intermediate concentrations. However, high doses of the drug induced cell death efficiently in both mock- and *BCL11B*-transduced cells (Fig. 3e). In contrast to DNA damage, apoptosis initiated by TRAIL treatment proceeded unperturbed regardless of *BCL11B* expression. Both the effector caspase 3 and the apical caspase 8 were efficiently activated in mock- and *BCL11B*-modified cells, in line with previously observed equal Annexin-V binding (Fig. 3f).

We next examined the influence of *BCL11B* expression on induction of DNA breaks resulting from etoposide and camptothecin treatment. The immunostaining for phosphorylation of histone H2AX (γH2AX), an early indicator of the cellular response to DNA damage, showed marked accumulation of γH2AX in cells with endogenous levels of Bcl11b while the cells with elevated Bcl11b showed only weak increase of γH2AX (Fig. 3g). In order to exclude the detection of apoptosis-related secondary DNA lesions, the measurements were performed before the first signs of apoptosis were detectable (Fig. 3g insets). Alternatively, to prevent the DNA breaks resulting from ongoing apoptosis, the same experiment was performed in the presence of pan-caspase inhibitor Z-VAD. Also under these conditions, *BCL11B*-overexpressing cells acquired less damage upon genotoxic stress (data not shown).

Together, these results suggested a role of *BCL11B* in preventing DNA-damaged-induced apoptosis at its early initiation stage. Conversely, the death-receptor triggered apoptosis remained unaffected.

### Bcl11b delays cell cycle progression

**Figure 4:**
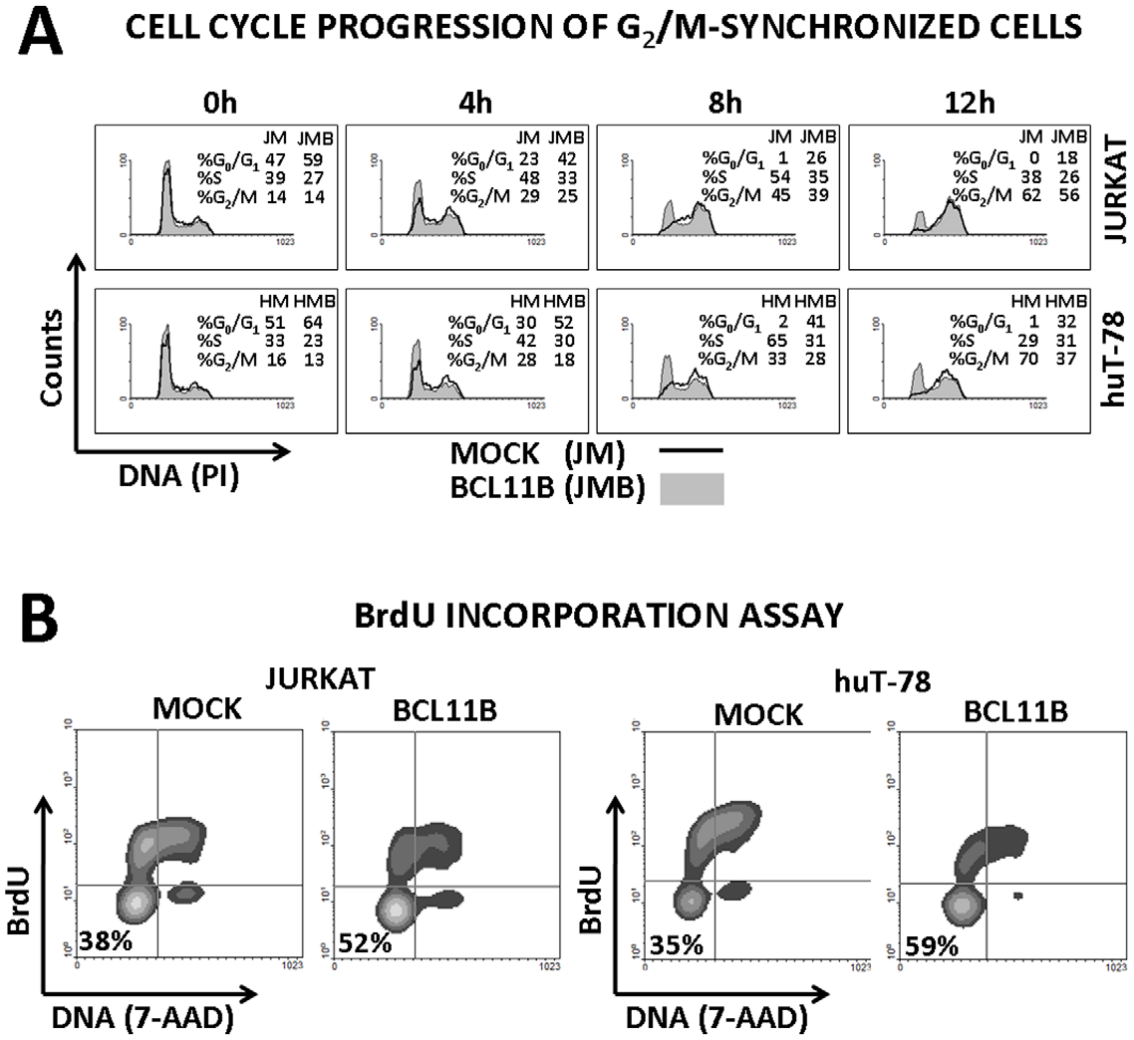
The impact of Bcl11b level on cell growth. (**A**) Jurkat and huT-78 cells with endogenous and enforced *BCL11B* expression were blocked at G_2_/M phase of cell cycle using mitosis inhibitor nocodazole (0,5 µM). The cell cycle distribution was evaluated at 0, 4, 8 and 12h by propidium iodide (PI) staining of genomic DNA followed by FACS measurement of PI fluorescence. (**B**) The mock- and *BCL11B*-transduced cells were incubated with the nucleotide analogue BrdU for 3h followed by fixation and permeabilization procedure. The incorporation of BrdU into genomic DNA reflecting the proliferative activity was demonstrated using a BrdU-specific, APC-labeled antibody (*y* axis). Total genomic DNA was stained with 7-aminoactimomycin D (7-AAD, *x* axis). These results are representative of at least three independent experiments.

The analysis of DNA content revealed that in both cellular models induction of *BCL11B* caused slight but significant accumulation of cells at G1 phase of cell cycle. To exclude the possibility that increased number of G1 cells resulted from accelerated cell divisions the cells were treated with a mitosis inhibitor nocodazol. The measurements performed after nocodazol treatment showed that while all mock-transduced cells entered cell cycle and reached either late S or G2/M phase within 12 hours, approximately 20–30% of cells with elevated Bcl11b levels remained at G1 (Fig. 4a). This was further demonstrated by the 3h pulse-labeling of the cells with the nucleotide analog BrdU followed by its immunodetection and FACS measurement. The number of BrdU-negative counts was significantly higher in *BCL11B*-transduced cells compared to cells with wild-type level of the gene (Fig. 4b). This suggested that forced expression of *BCL11B* caused either G1 arrest or a delayed G1 to S-phase transition in a fraction of cells.

**Figure 5:**
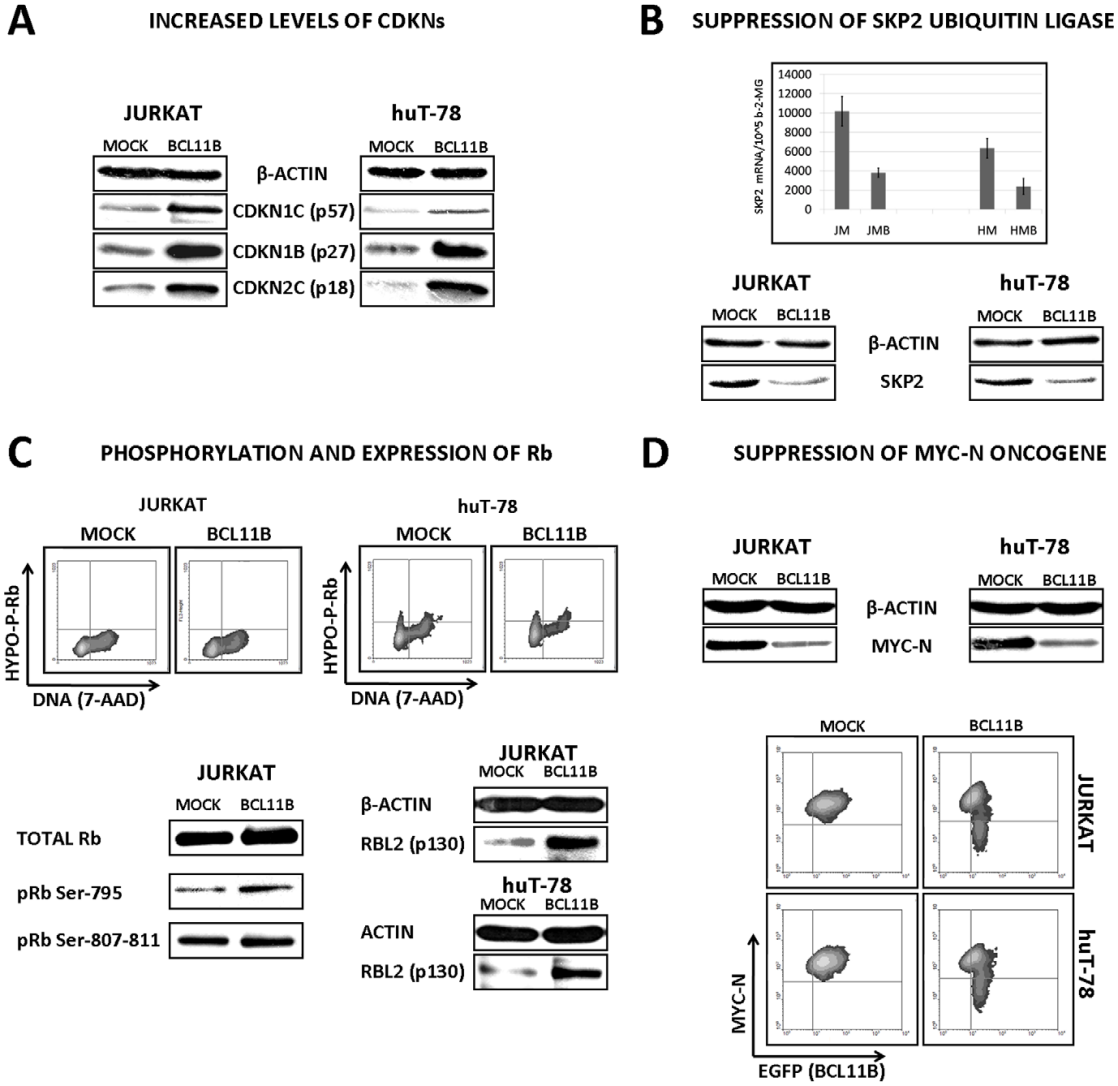
Regulation of cell cycle-controlling genes following Bcl11b accumulation. (**A**) The protein lysates acquired from mock- and *BCL11B*-transduced Jurkat and huT-78 cells were analyzed by Western blotting using indicated antibodies 48h post-transduction. (**B**) The effects of *BCL11B* overexpression on ubiquitin ligase *SKP2* was evaluated by semi-quantitative RT-PCR and western blot 48h after transduction. (**C**) The retinoblastoma protein phosphorylation status was assayed using antibodies preferentially binding to hypophosphorylated variant of pRb followed by flow cytometry. Additionally, the antibodies recognizing specifically the phosphorylated pRb and total retinoblastoma-like protein 2 were used to detect the proteins by western blot. (**D**) The amount of Myc-N protein in cells with normal and elevated Bcl11b levels were assessed by immunodetection in protein lysates (upper part, western blot). In addition, a Myc-N-specific antibody was used for intracellular staining followed by flow cytometry (lower panel, density plot shows reporter gene – X axis, and Myc-N – Y axis). All western blot and FACS data shown on this figure represent results obtained from at least three experiments. Section B (upper part, semi-quantitative RealTime RT-PCR) shows average values from three experiments ± SD, *p<0.05.

The whole-genome expression profiles of mock- and *BCL11B*-transduced Jurkat cells uncovered the regulation of genes known to be involved in cell cycle control (GEO accession number: GSE21382). The list of genes verified by semi-quantitative RT-PCR (sqRT-PCR) is presented in Table 1. Our attention focused on genes with previously confirmed function in G1 or G1 to S-phase transition. First, the induction of two cyclin dependent kinase inhibitors *CDKN1C* (p57) and *CDKN2C* (p18) was confirmed on protein level by Western Blotting in both experimental models (Fig. 5a). While verifying the involvement of the other members of this gene family we found significant accumulation of *CDKN1B* (p27) which resulted either from increased stability or defective degradation since no major increase of p27 mRNA could be measured (data not shown). The later possibility seemed more likely since the transcriptional downregulation of the *SKP2* gene involved in proteasomal degradation of p27 was identified. Moreover, the level of Skp2 declined markedly upon *BCL11B* induction (Fig. 5b).

**Table 1:** Cell cycle-related genes identified as at least 2-fold regulated after *BCL11B* overexpression in Jurkat cells using Affymetrix Arrays and confirmed by quantitative RT-PCR in independent experiment in two different cellular systems.

Symbol	Name	Fold change (Affymetrix Array)	Fold Change (qRT-PCR) in Jurkat	Fold Change (qRT-PCR) in huT-78
*MYCN*	v-myc myelocytomatosis viral related oncogene, neuroblastoma derived (avian)	−2.0	−2.4	−3.3
*BCL6*	B-cell CLL/ Lymphoma 6	3.0	3.7	2.5
*CCNG2*	Cyclin G2	2.9	2.4	4.7
*CDKN1C*	Cyclin-dependent kinase inhibitor 1C	2.1	3.5	2.7
*CDKN2C*	Cyclin-dependent kinase inhibitor 2C	2.1	3.8	3.0
*HBP1*	HMG-box transcription factor 1	2.6	2.7	2.2
*TP53INP1*	Tumor protein p53 inducible nuclear protein 1	8.0	6.4	5.2

Interestingly, in Jurkat cells we did not find any differences in pRB phosphorylation status which usually precedes the cell cycle entry. This was confirmed by using phospho-specific antibodies labeled with fluorescent dyes followed by FACS analysis in the other cellular system (Fig. 5c). The level of retinoblastoma-like protein 1 (*RBL1*, p107) did not vary either (not shown). However, the third member of this gene family *RBL2* (p130), normally undergoing degradation during cell cycle entry, showed significantly increased protein levels (Fig. 5c). This suggested the possible involvement of pocket proteins and their target genes – E2Fs in cell cycle delay resulting from *BCL11B* induction. This assumption was further strengthened by the 3-fold reduction in the level of the *MYCN* oncogene which is negatively regulated by Rbl2 [30]. The decreased *MYCN* transcription was confirmed in *BCL11B*-overexpressing cells in both experimental models by sqRT-PCR and verified on protein level by Western blotting in Jurkat cells. Moreover, the detection of the Myc-n protein by immunofluorescence followed by FACS revealed a negative correlation between the Myc-n signal and the intensity of the reporter gene formerly shown to correspond to Bcl11b levels (Fig. 5d). This indicated that elevated Bcl11b, apart from inducing p18, p27 and p57 could delay the cell cycle entry by causing accumulation of RBL2 which suppressed *MYCN* expression. The upregulation of the *HBP1* transcription factor (Table 1.) known to cooperate with *RBL2* in suppressing the *MYCN* promoter [30] strongly implied the potential involvement of the Bcl11b-p130/Hbp1-Myc-n axis in the observed G1 arrest/delay.

In sum, we revealed potential mechanisms of Bcl11b-mediated cell cycle restriction which included activation or induction of p18 and p57 cyclin dependent kinase inhibitors, accumulation of p27 p130 and suppression of *MYCN* oncogene. The increase of cells at G1 phase and decreased fraction of replicating cells could explain the low toxicity of radiomimetic treatment with etoposide and camptothecin.

### Cell cycle restriction and DNA damage resistance caused by *BCL11B* overexpression require its HDAC-interacting N-terminal domain

**Figure 6:**
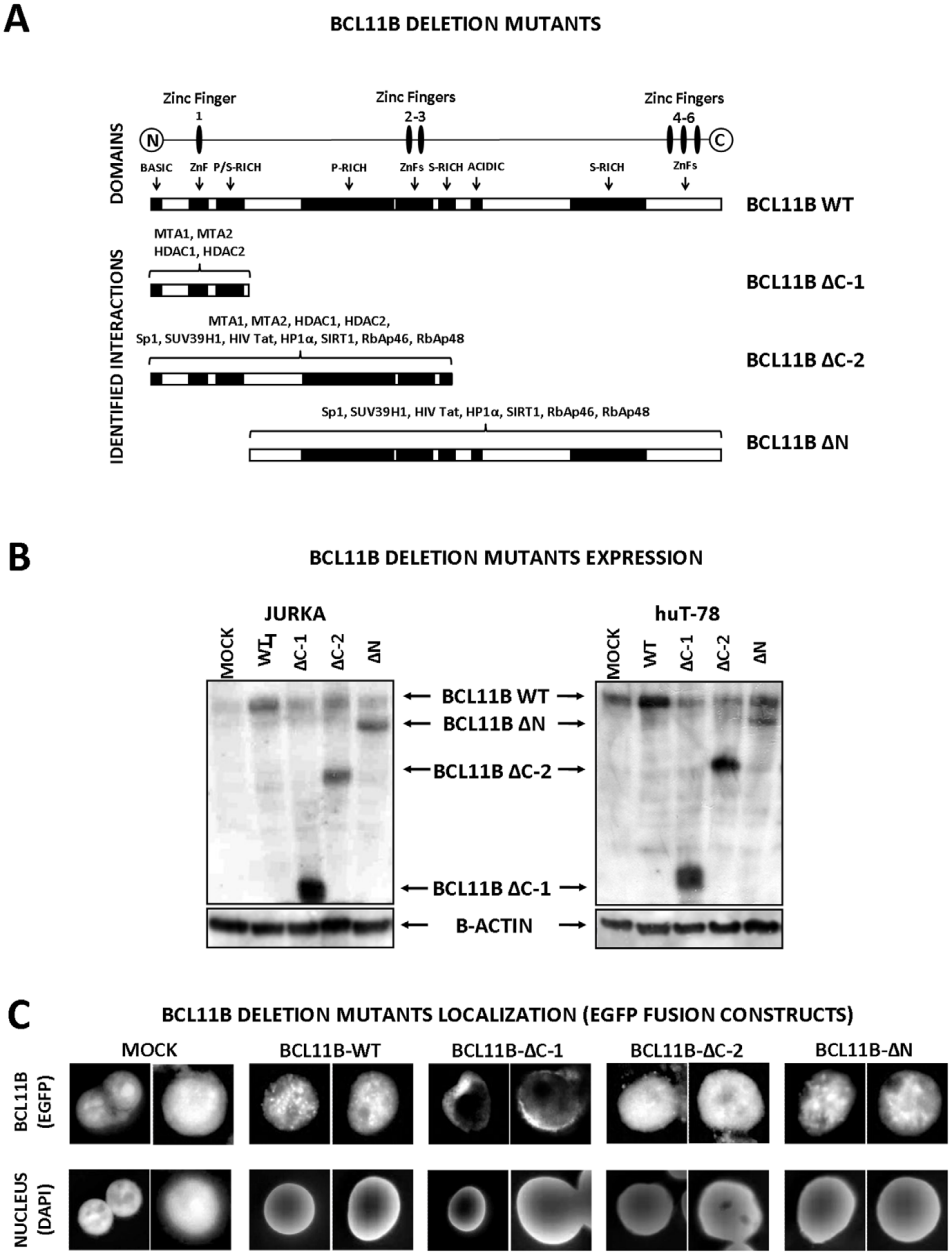
Bcl11b deletion mutants: design, expression and localization. (**A**) Schematic diagram of Bcl11b structure and the *BCL11B*-derived deletion mutants; the characteristics of the conserved domains (black boxes) are indicated. Abbreviations: ZnF(s) – zinc finger(s), P-rich – proline- and S-rich – serine-rich regions. The cooperating proteins are listed above the interacting domains included in the truncated *BCL11B* variants; HDAC1 and 2 – histone deacetylases 1 and 2, MTA1 and 2 – metastasis-associated gene 1 and 2, Sp1 – transcription factor Sp1, SUV39H1 – suppressor of variegation 3–9 (homolog of), HIV Tat – human immunodeficiency virus Tat protein, HP1α – heterochromatin protein 1, Sirt1 – sirtuin 1, RbAp46 – retinoblastoma-binding protein 7, RbAP48 – retinoblastoma-binding protein 4. (**B**) Jurkat and huT-78 cells were transduced with retroviral vectors encoding wild-type *BCL11B* and the *BCL11B* deletion mutants. The expression of the transgenic proteins was verified by western blot and subsequent immunodetection using the cocktail of Bcl11b-specific antibodies targeting N-, C-terminal and internal epitopes of the protein. The β-actin protein level was analyzed to confirm equal protein loading. (**C**) DNA fragments encoding whole-length and truncated variants of *BCL11B* were cloned in-frame into the expression vector downstream of EGFP gene. The vectors were transfected into Jurkat cells and the sub-cellular localization of the fusion proteins was analyzed by fluorescent microscopy (upper panel). The nuclei were counterstained with the DNA-specific dye DAPI (lower panel).

Bcl11b was identified as a part of several multiprotein complexes and a number of Bcl11b domains involved in these interactions have been identified. Therefore, we were interested in identifying the region of Bcl11b relevant for binding complexes/proteins necessary for the DNA-damage resistance and/or cell cycle restriction following its overexpression. We designed, cloned and expressed three different *BCL11B* variants covering the regions known to be important for the formerly established interactions/functions (Fig. 6a.). The C-terminally truncated variant one encoded the NuRD interacting N-terminal domain (*BCL11B* ΔC-1), the longer variant 2 contained additionally the SUV39H1/HP1/SIRT1/Sp1 /Tat binding domain consisting of the proline-rich region and two central zinc fingers (*BCL11B* ΔC-2). The third mutant allele contained the whole coding sequence excluding the N-terminal part (*BCL11B* ΔN). The constructs were used for transduction of Jurkat and huT-78 cells and the protein expression and the calculated molecular weight was confirmed by Western blot analysis (Fig. 6b.). To test the sub-cellular localization the deletion mutants were sub-cloned in frame into the pEGFP-C1 vector and transfected by nucleofection into Jurkat cells. As shown on Fig. 6c, the transfection of EGFP-only vector (mock) caused even distribution of green fluorescence protein within the cells. In contrast, when fused to wild-type *BCL11B* cDNA the EGFP was located in the nucleus showing the spackle-like structures previously described by others. A pattern similar to the wild-type pattern could be detected in cells transfected with *BCL11B* ΔC-2 and *BCL11B* ΔN deletion mutants. Contrary, the N-terminal part (*BCL11B* ΔC-1) localized in the extranuclear compartment. These data indicated that the nuclear localization signal was located downstream of NuRD-interacting N-terminal domain and its deletion caused aberrant non-physiological location of the fusion protein.

**Figure 7:**
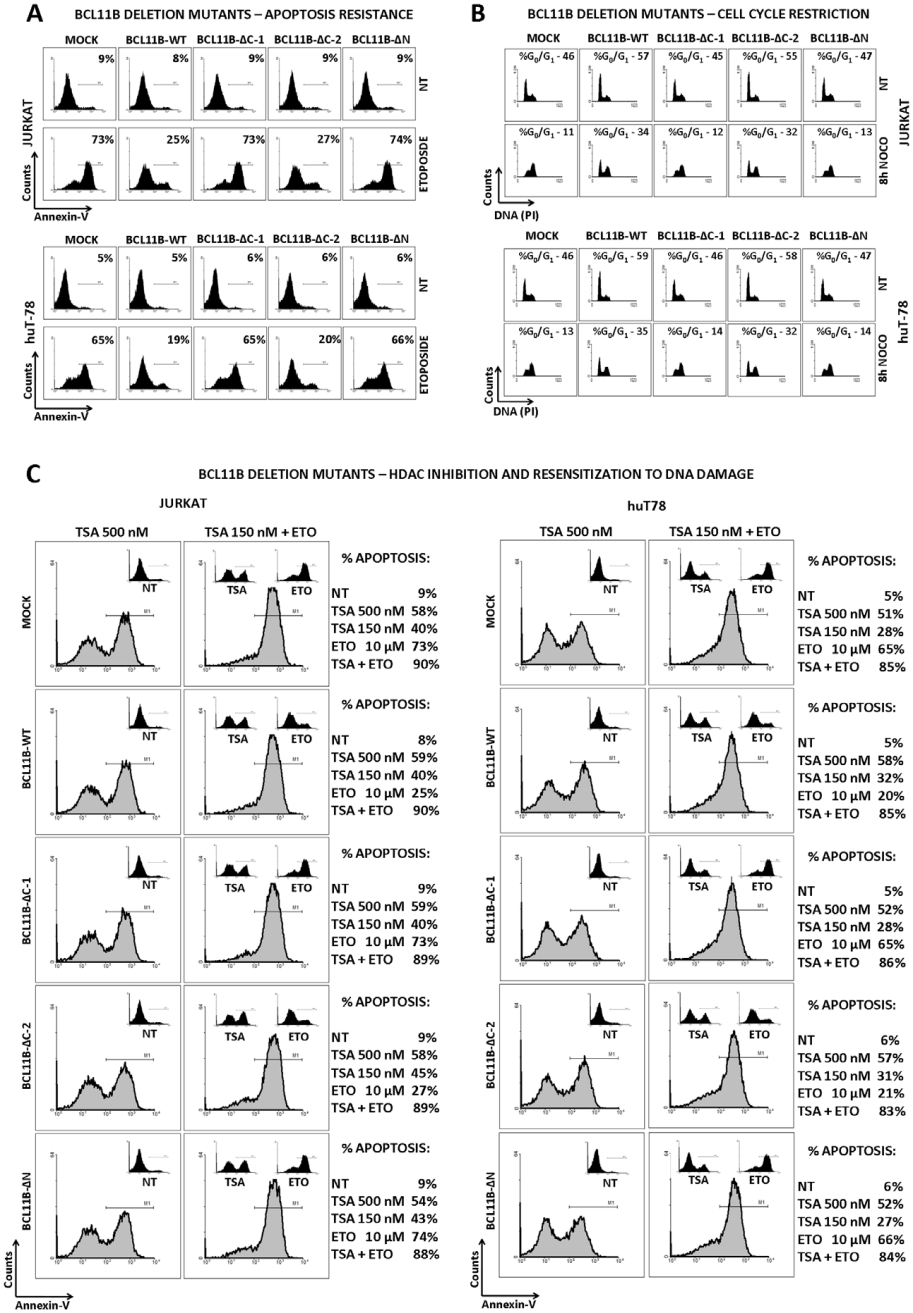
Bcl11b truncated mutants: apoptosis resistance, cell cycle retardation and HDAC dependence. (**A**) Jurkat and huT-78 cells transduced with the empty vector (mock) and constructs encoding the full-length or truncated *BCL11B* variants were exposed to etoposide for 6h after which the viability was analyzed by Annexin-V binding assay. (**B**) The T cell lines expressing endogenous (mock), enforced (*BCL11B*-WT) and truncated *BCL11B* (Δ) were synchronized at G_2_/M phase of cell cycle using mitosis inhibitor nocodazole (0,5 µM). The effect of elevated Bcl11b and Bcl11b-derived mutant proteins on cell cycle was analyzed before and 8h after initiation of nocodazole treatment. The cell cycle distribution was analyzed by propidium iodide (PI) staining of genomic DNA followed by FACS measurement of PI fluorescence. (**C**) Jurkat and huT-78 cells transduced with empty or *BCL11B*-encoding vectors (wild-type and truncated) were treated for with high dose of histone deacetylase inhibitor trichostatin A (TSA, left panels) or left untreated (insets). In addition, a lower dose of TSA (150 nM) was combined with the standard etoposide treatment (right panels). The treatments with TSA- and etoposide-only were performed for comparison (insets). The effects of the drugs on the viability of cells expressing endogenous, elevated and truncated Bcl11b was measured with Annexin-V binding assay followed by FACS quantification. The data displayed here represent results obtained from one of at least three separate experiments.

Next we investigated whether and to which extent the N- and C-terminally truncated *BCL11B* variants preserved the properties of wild-type gene. The induction of DNA damage by the radiomimetic drug etoposide in cells transduced with empty vector, wild-type and truncated *BCL11B* alleles followed by apoptosis assays confirmed the protective activity of wild-type *BCL11B* in both transduced T-cell lines (Fig. 7a.). As expected, the aberrantly located *BCL11B*- ΔC-1 variant did not have any influence on DNA-damage induced apoptosis. Conversely, the second C-terminal mutant *BCL11B*- ΔC-2 lacking almost half of the protein including three zinc fingers was as effective in preventing cell death as its wild-type counterpart. The removal of the N-terminal domain (*BCL11B*- ΔN) in turn resulted in the complete loss of anti-apoptotic properties. Since we previously showed that the DNA-damage resistance was accompanied by and presumably associated with cell cycle restriction at the pre-replication phase, we performed the synchronization of the transduced cells at G_2_/M using nocodazol (Fig. 7b.). DNA staining before nocodazol treatment showed a slightly elevated G_0_/G_1_ fraction in *BCL11B*- ΔC-2 and wild-type *BCL11B* transduced cells. Eight hours later the accumulation at late S and/or G_2_/M phases and essentially no G_0_/G_1_ was observed in cells transduced with empty, *BCL11B*- ΔC-1 and *BCL11B*- ΔN-containing vectors. However, *BCL11B*- ΔC-2 and wild-type *BCL11B* overexpression caused a significant block of cell cycle at G_0_/G_1_ which correlated well with the DNA-damage resistance. These results indicated that the anti-apoptotic and cell cycle regulatory activities of BCL11B are strictly dependent on the presence of the N-terminal region, previously reported to interact with protein complexes containing histone deacetylases (HDAC1 and 2). To verify the involvement of HDACs in Bcl11b-mediated cell cycle control and low sensitivity to genotoxic stress we applied the histone deacetylase inhibitor trichostatin A (TSA). At high dose, TSA showed equal toxicity to mock-, *BCL11B*-WT and *BCL11B*-mutant-transduced cells as measured by Annexin V-binding assay (Fig. 7c.). The sub-optimal dose of TSA caused generally lower but comparable toxicity (Fig. 7c insets). Besides this, the low doses of TSA re-sensitized the cells transduced with *BCL11B* and *BCL11*B-ΔC-2 mutant to etoposide treatment (Fig. 7c). Similar results were obtained with a different HDAC class I and II inhibitor SAHA, while a class III inhibitor, blocking SIRT1 activity was ineffective in interfering with Bcl11b mediated protection (data not shown). This provided further evidence for the engagement of HDAC1 and 2 in genotoxic stress resistance and cell cycle restriction observed upon induction of *BCL11B*. Of note, the N-terminal, HDAC-binding region itself was not sufficient for the described effect. Even when physiologically localized in the nucleus *via* addition of the SV40 nuclear localization signal at its C-terminus it did not alter the DNA-damage sensitivity or cell cycle progression (not shown).

## Discussion

The crucial role of *BCL11B* gene in the regulation of cell cycle and apoptosis has been postulated based on the results obtained from both *in vitro* and *in vivo* studies. However, its potential positive or negative contribution to tumor development was not convincingly settled so far. The growth-promoting properties of truncated *BCL11B* variants isolated from leukemic cells [20], the losses of heterozygosity at the very early stages of lymphomagenesis [31] and detection of *BCL11B* haploinsufficiency for suppression of mouse thymic lymphomas [29], [32] support the speculation of *BCL11B* tumor suppressive activity. On the other hand, in the majority of human T-ALLs the expression of *BCL11B* was similar to the level obtained by our overexpression approach in Jurkat and huT-78 (Fig. 1B). Moreover, amplifications of the *BCL11B* locus accompanied by increased mRNA expression and protein were shown in adult T cell leukemia/lymphoma and correlated with the aggressiveness of the disease [33] which indicates a function opposite to tumor suppression. Diverse Bcl11b features can be observed also on the biochemical level. Sharing structural similarities with transcriptional repressors [34] and interacting with chromatin-silencing protein complexes [35], [36], [37], Bcl11b can act also as a potent transcriptional activator [10], [12]. The list of the genes negatively regulated by Bcl11b contains the known tumor suppressors p57/*KIP2* [38] and p21/*WAF1* [13], while the activated targets include genes inducing and regulated by NFκB, like Cot kinase or *IL-2*, respectively [10], [12]. These data, supported by the observed elevated expression of *BCL11B* in T cell malignancies, imply its pro-survival and growth-promoting function. The notion was strengthened by findings showing the critical dependence of transformed T cells' survival on *BCL11B* expression [22], [23].

In summary, *BCL11B* seems to function pleiotropic and may lead to opposite effects in different cellular systems. This encouraged us to evaluate the consequences of the increased *BCL11B* levels in transformed cells endogenously expressing the gene. We developed a retrovirus-mediated *BCL11B* overexpression system in two different T cell leukemia cell lines and focused our research on apoptosis and proliferation. We found significantly increased resistance of *BCL11B*-overexpressing cells to genotoxic-stress and cell death which appeared to be blocked at the early stages of the process. Conversely, apoptosis triggered by death receptors remained undisturbed suggesting that the enhanced survival upon genotoxic stress is not due to a general apoptosis failure. Interestingly, global RNA profiling of wildtype and *BCL11B*-overexpressing cells did not reveal any significant differences in the expression of genes known to be engaged in the canonical cell death pathways. The immunodetection of the pro- and anti- apoptotic members of the BH3-only protein family in non- or etoposide treated mock- and *BCL11B*- transduced cells also did not show any major increase of the anti-/pro-apoptotic protein ratio (data not shown). The observed lower number of DNA lesions in *BCL11B*-overexpressing cells upon damage induction suggests limited efficiency of the DNA-damaging drugs as the likely reason for the lower death rate in *BCL11B*-overexpressing cells. In light of the previously described growth-limiting effect of *BCL11B* in *BCL11B*-negative HeLa and hematopoietic progenitor cell FDC-P1lines, a tempting hypothesis is that cell cycle restriction causes the reduced percentage of S-phase cells. Since the DNA-damaging treatment we applied was proven to kill predominantly cells in S-phase [39] the diminished fraction of cells replicating DNA resulting from G1 accumulation would lead to the chemoresistance of the non-replicating cell population. We confirmed this possibility by pre-treating Jurkat and huT-78 cells with the DNA polymerase inhibitor aphidicolin which reduced the rate of G1 to S transition and limited the fraction of S phase cells. When subsequently treated with etoposide, camptothecin or other radiomimetic drugs a fraction of cells blocked at G0/G1 phase cells remained vital while the dead cells originated from S phase (data not shown). The cell cycle analysis and bromodeoxyuridine (BrdU) incorporation assays showed a clear decrease of the S phase fraction in both cell lines upon *BCL11B* overexpression compared to mock treatment. This was due to G1 arrest which was most evident when the cells were synchronized at G2/M with nocodazol which explains the lower sensitivity to radiomimetic drugs. Of note, the G1 arrest was not permanent. When pulse-treated with etoposide or other DNA-damaging drugs, the *BCL11B*-transduced cells restored growth after several days while the mock-transduced cells died. This observation suggests that elevated levels of Bcl11b could trigger a fraction of chemoresistant tumor-initiating cells. Surprisingly, the cell cycle arrest seemed not to be accompanied by the activation of the checkpoint mechanism and the phosphorylation status of the checkpoint proteins Chk1/Chk2 was not significantly altered (not shown). This indicates that although the lack of Bcl11b resulted in checkpoint inactivation [23], the cell cycle delay caused by *BCL11B* overexpression was mediated by a Chk1/Chk2 checkpoint-independent mechanism. The candidate genes potentially involved in the observed cell growth suppression were identified by the comparison of mRNA profiles of mock- and BCL11B-transduced cells. This analysis revealed upregulated expression of the G1 cycline-dependent kinase inhibitor *CDKN2C* (p18/Ink4c), leading to elevated levels of the encoded protein. The p18 protein binds and inhibits selectively two cyclin dependent kinases, Cdk4 and 6, which are responsible for controlling G1 to S transition. The ectopic expression *CDKN2C* suppresses cell growth in a manner resembling the activation of the tumor suppressor gene pRB [40].

Another representative of the CDKis found to be upregulated in *BCL11B*-transduced cells is *CDKN1C* (p57/Kip2). The protein encoded by this gene is a potent, tight-binding inhibitor of several G1 CDKs, arresting the cells in G1 when overexpressed [41]. The induction of this gene in cells with elevated Bcl11b is especially interesting since the gene has been previously shown to be negatively regulated by Bcl11b *via* associating the NuRD nucleosome remodeling complex [38]. A potential mechanism which could explain the observed transcriptional activation and accumulation of p57 could be based on the “titration” model, in which the accessibility of HDACs necessary for suppression is limited by an excess of Bcl11b. A depleted activity of HDACs as a cause of p57 induction was already identified. Different class I and II HDAC inhibitors showed a potent inducing activity on *CDKN1C*. As shown recently, a different member of the Krueppel-like factor family KLF4 demonstrated a potent anti-apoptotic effect upon HDACi treatment which was mediated in part by direct binding and transcriptional upregulation of p57 followed by inhibition of the stress response phosphorylation pathway [42]. Finally, the role of E2F1 in the transcriptional regulation of p57 must be considered. We report here the activation of the Cdk 4/6 inhibitor p18. As communicated recently, the inhibition of the cyclin dependent kinases by small molecules led to the transcriptional activation of *CDKN1C* which involved direct binding of E2F1 to the promoter. It was suggested that this regulatory loop served to limit the E2F1's death-inducing activity [43].

The third member of the cyclin-dependent kinase inhibitors which accumulated in BCL11B-overexpressing cells was *CDKN1B* (p27/Kip1). It was previously reported that downregulation of Bcl11b led to the transcriptional suppression and decrease of p27 protein [23], [44] suspected of being one of the cell-death triggering events after *BCL11B* knockdown. This, together with our finding implied a correlation between Bcl11b and p27 levels. However, we did not observe an effect of elevated Bcl11b on p27 transcription indicating the involvement of a post-transcriptional regulation. The level of p27 protein can be modulated *via* multiple posttranscriptional mechanisms [45]. The most potent and the best characterized mechanism of p27 regulation is its degradation preceded by polyubiquitylation by the SCF^SKP2^-E3 ubiquitin ligase complex at G1 phase [46]. The involvement of the impaired Skp2-E3-mediated p27 proteolysis in our system is strengthened by the fact that *SKP2* was significantly reduced on mRNA and protein level in cells transduced with *BCL11B*. Support for functional relevance of the decreased Skp2 level following *BCL11B*-transduction was provided by accumulation of another substrate of the Skp2-E3 complex, the retinoblastoma-like protein 2 (*RBL2*/p130) which was not accompanied by any transcriptional activation [47]. The increase of p130 protein was the only change that could be identified within the pocket protein family known to be crucial for G1 to S transition. However, the accumulation of p130 could contribute to cell cycle retardation and apoptosis resistance in *BCL11B*-transduced cells. In neurons, the integrity of the E2F4-p130-HDAC/SUV39H1 complex was shown to be critical for cell survival and overexpression of p130 significantly increased the apoptosis resistance triggered by camptothecin treatment [48]. Furthermore, p130 participated in the suppression of the *MYCN* promoter by interacting with the HBP1 high mobility group (HMG) transcription factor increased transcription of which was observed in *BCL11B*-overexpressing cells, [30]. The involvement of the described mechanism in our experimental system was supported by markedly decreased *MYCN* transcription upon BCL11B-transduction. In the cells expressing *BCL11B* at higher levels as visualized by the strong EGFP signal, Myc-N protein was almost undetectable, while the cells with dim green fluorescence showed stronger signals. This observation argues for a strict negative correlation between Bcl11b and Myc-N. Interestingly, inactivation of *MYCN in vivo* led to the activation of p27/Kip1 and p18/Ink4c [49] which indicates that suppression of *MYCN* expression could lead to the effects triggered by *BCL11B* overexpression. However, it seems that changes in the level of Myc-N are not the sole reason for cell cycle abnormalities and cell death resistance. Overexpression of *MYCN* markedly increased proliferation of Jurkat and huT-78 cells expressing endogenous levels of *BCL11B* but only minimally influenced the cell cycle progression in cells transduced with *BCL11B*-encoding vector (data not shown). The notion that parallel activation of multiple pathways seems to lead to the growth suppression and apoptosis resistance observed after BCL11B overexpression is also supported by additional studies on CDKis. Neither the specific knockdown of the particular CDKi nor the simultaneous suppression of two or three CDKis could restore normal growth or apoptosis resistance.

In order to get more insight into potential mechanisms of Bcl11b-mediated cell cycle and cell death regulation, we created a series of *BCL11B* deletion mutants lacking the important DNA-binding and protein-interacting domains. Our data strongly indicate that the identified biological consequences of *BCL11B* upregulation were strictly dependent on the presence of the N-terminal HDAC-interacting domain. The removal of the C-terminal half of *BCL11B* cDNA, encoding the three zinc fingers but carrying the N-terminal domain retained the function of wild-type *BCL11B*. Conversely, the deletion of the N-terminal part completely inactivated the mutant protein, although it localized physiologically in the nucleus. Interestingly, the short fragment encoding just the HDAC-interacting region did not show any *BCL11B*-resembling activity, even when localized in the nucleus by addition of viral NLS. Of course one cannot eliminate the improper folding of such a truncated protein as the reason of its inactivation.

Taken together, it appears that Bcl11b acts by recruiting some factors to the DNA template through its HDAC-binding domain. In order to provide additional support for this hypothetical mechanism, we treated wt and *BCL11B*-overexpressing cells with the HDAC inhibitor trichostatin A (TSA). Unfortunately, we could not investigate the effects of TSA on cell cycle arrest upon *BCL11B*-transduction because of high toxicity of the drug alone and in combination with the G2/M synchronizing nocodazol. However, we observed a significant re-sensitization of TSA-treated *BCL11B*-overexpressing cells to DNA-damage induced apoptosis, supporting the involvement of HDAC in Bcl11b-mediated cell cycle and apoptosis control.

Our results suggest that *BCL11B* may perform opposite functions depending on the cellular context and the expression level. The upregulation of the gene significantly increases the genotoxic stress resistance which could constitute an oncogenic feature providing chemoresistance and allowing the survival of transformed cells. On the other hand, the insensitivity to DNA damage is accompanied by a markedly lower proliferative activity, which is considered to be tumor suppressive. We speculate that in non-transformed tissue *BCL11B* could control the proliferation, ensure genome stability and prevent tumor development and/or apoptosis. This feature qualifies *BCL11B* as a tumor suppressor or a gene maintaining the balance between proliferation and cell death. But in the context of a tumor tissue *BCL11B* could act as potential oncogene being an attractive target for therapeutic approaches.

Similar ambivalent modes of action were identified recently for p21/*WAF1* in a mouse model of leukemia [50]. In this setting a prototype tumor suppressor manifested its oncogenic features by restricting cell cycle followed by limited DNA damage and consequently maintaining self-renewal potential of leukemic stem cells. Of note, *BCL11B* was found to be co-expressed with the cancer stem cell marker *BMI-1* in a subset of undifferentiated squamous cell carcinoma cases and cell lines [27]. This indicates that elevated *BCL11B* could play a similar role in tumors expressing it by controlling proliferation and chemoresistance of tumor-initiating cells. The survival assay presented here seems to confirm such a possibility. Jurkat and huT-78 cell forced to overexpress *BCL11B* survived and efficiently restored cell growth upon temporal exposition to genotoxic stress.

In conclusion, we believe that the data presented here strengthen the role of *BCL11B* in tumor survival rather than development. Targeting Bcl11b *via* inhibitory approaches might be a promising tool for the treatment of rapidly growing list of cancer types expressing *BCL11B*. Moreover, the identification of HDAC-interacting domain of Bcl11b as a critical mediator of its activity and the finding that BCL11B-mediated apoptosis resistance can be reversed by HDACi may have important implication for therapeutic interventions against *BCL11B*-positive tumors.

## Materials and Methods

### Reagents

All biochemicals were from Sigma Chemicals (Munich, Germany) unless otherwise specified. Human recombinant TRAIL was purchased from PeproTech (Peprotech Ltd, London, United Kingdom). The Bcl-2/BCLxL inhibitor ABT-737 was provided by Abbott (Abbott Laboratories Il, USA).

### T-cell leukemia samples

The 20 mRNAs isolated from T-cell lymphoblastic leukemias were provided by different clinical units. All samples were obtained according to the guidelines for informed consent approved by local Ethical Research Committees.

### Plasmids

The constructs used in this study were based on the pMSCV-derived retroviral vector pMIGR encoding internal ribosome entry site followed by EGFP gene. The sequence encoding the full length *BCL11B* was amplified from cDNA prepared from healthy individual and cloned between *Bgl*II and *Eco*RI restriction sites. The deletion mutants of *BCL11B* were constructed using PCR techniques with proof reading polymerase Pfu (Promega, WI, USA ) using primers equipped with *Bgl*II and *Eco*RI recognition sequences and start/stop codons where required. All constructs were checked by sequencing to exclude the presence of mutations. The vector carrying the pantropic viral envelope protein pVSV-G was purchased from Clontech (Clontech Laboratories, CA, USA).

### Cell culture, transfection and transduction

The human T cell leukemia and lymphoma cell lines Jurkat (DMSZ, Braunschweig, Germany) and huT-78 (ATCC, Rockland, MD) were maintained in RPMI-1640 medium supplemented with 10% fetal calf serum (PanBiotech Berlin, Germany), Glutamax (Invitrogen, CA, USA) and Plasmocin (Lonza Scientific, Switzerland). The retrovirus producing cell line GP2-293 (Clontech Laboratories, CA, USA) was maintained in Dulbecco's modified Eagle's medium (Invitrogen, CA, USA) supplemented as above.

For recombinant retrovirus production, non-confluent GP2-293 cells were co-transfected with 10 µg of purified pVSV-G and empty pMIGR plasmid (mock) or pMIGR encoding *BCL11B* variants. The transfection procedure was performed using CalPhos Mammalian Transfection Kit (Clontech Laboratories, CA, USA) according to the manufacturer's instructions. The retrovirus-containing media were collected 48h after transfection and used immediately or stored at 4°C and used within 7 days. To transduce the target cells, the retroviral supernatants were supplemented with 8 µg/ml Polybrene (Sigma Chemicals, Germany) and added to exponentially growing Jurkat and huT-78 cells for 8 hours. The procedure was repeated three times. The efficiency of gene transfer was estimated by FACS detection of EGFP reporter gene. To ensure high proportion of transduced cells, EGFP-positive cells were enriched using high-speed cell sorter FACSAria (BD Biosciences, NJ, USA). Sorted, at least 90% EGFP-positive cells were used for further experiments.

### Apoptosis induction and viability assays

To induce DNA-damage, Jurkat and huT-78 cells transduced with the empty- and *BCL11B*-encoding retroviral vectors were treated with etoposide, camptothecin, actinomycin D or dihydroethidium (Sigma, Germany). The influence of histone deacetylase inhibition on the viability of mock- and *BCL11B*-modified cells was verified using increasing concentrations of trichostatin A, nicotinamide (Sigma, Germany) or SAHA (Cayman Chemicals, MI, USA). To investigate the role of histone deacetylases in DNA damage resistance of *BCL11B*-overexpressing cells, the treatment was combined with damage induction using etoposide. Death-receptor pathway was activated by TRAIL (ProImmune Ltd., United Kingdom). Where indicated, incubation with etoposide was combined with the ABT-737 Bcl-2/BCLxL- inhibitor treatment (Abbott Laboratories, Il, USA). The long term survival upon genotoxic stress was initiated by 6 hours pulse treatment with radiomimetic drugs followed by extensive washing and 14 days cell culture accompanied by cell counting every 48 hours. Apoptosis was measured by FACS using Annexin V-APC binding assay (BD Pharmingen, CA, USA). The activation of caspase 3 and 8 was quantified by immunofluorescent detection of the cleaved variants followed by FACS analysis. In brief, mock- and *BCL11B*-transduced Jurkat and huT-78 cells were fixed/permeabilized using Cytofix/Cytoperm kit (BD Pharmingen, CA, USA) and incubated with cleaved caspase 3 (Asp175) or cleaved caspase 8 (Asp391) rabbit monoclonal antibodies and subsequent staining with the secondary goat anti-rabbit IgG F(ab)^2^ fragment stained with APC (Santa Cruz Biotechnology, CA, USA). Activation of caspase 9 was assayed by western blot using rabbit polyclonal antibody recognizing the cleaved/active variant of the protein (Cell Signaling Technology, MA, USA). The integrity of mitochondria was evaluated with the live-mitochondria-specific dye Mitotracker Deep Red FM (Invitrogen, CA, USA) and subsequent FACS measurements. Activation of Bak1 pro-apoptotic protein was tested by immunofluorescence using anti-Bak mouse monoclonal antibody (clone TC-100, Calbiochem, Merck Chemicals, Germany) binding the N-terminal domain exposed upon protein activation and the secondary goat anti-mouse-APC antibody. The determination of the DNA damage induced by radiomimetic drugs was performed by immunofluorescence using AlexaFluor 647-labeled anti-phospho-histone H2A.X antibody (γH2A.X, Cell Signaling, MA, USA) two hours upon treatment initiation. The relative signals obtained in mock- and *BCL11B*-transduced Jurkat and huT-78 cells were measured by FACS. The progress of apoptosis was monitored at the same time by Annexin-V binding assay. To further exclude the secondary, apoptosis related DNA breaks, the same procedure was repeated in the presence of 20 µM pan-caspase inhibitor Z-VAD (Sigma, Germany).

### Cell cycle analysis

Cell cycle status has been determined by propidium iodide DNA staining and flow cytometry analyses. Briefly, the cells were washed and resuspended in propidium iodide staining solution containing RNase A (Sigma, Germany) and incubated in the dark for 30 min. Flow cytometric analysis was performed in a FACScalibur instrument (Becton Dickinson, San Jose, CA, USA). Cell cycle analysis was performed using ModFit *LT* (Verity Software House, Topsham, ME, USA). To determine the rate of G1 to S transition, cells were blocked in G2/M phase with 0,5 µM nocodazole and the cell cycle analysis was done at different time-points. In addition, the G1 to S progression was assessed by pulse treatment with the nucleotide analogue BrdU. BrdU incorporation, reflecting the speed of cell cycle progression was determined with a fluorescently labeled antibody and measured by flow cytometry (APC BrdU Flow Kit, BD Pharmingen, Franklin Lakes, NJ, USA).

### RNA isolation, microarray analysis and quantitative RT-PCR

Total RNA was isolated from Jurkat cells transduced with empty and *BCL11B* encoding vector with Trizol Reagent (Invitrogen). Affymetrix array analysis was performed using the One-Cycle Target Labelling and Control Reagents, which contain the GeneChip Sample Cleanup Module, and Human Genome U133 Plus 2.0 DNA arrays (Affymetrix, Santa Clara, CA) according to the manufacturer's instructions. The subsequent scanning was performed with the GeneChip Scanner 3000 (Affymetrix). For quantitative RT-PCR (qRT-PCR), RNA was reverse transcribed with MultiScribe reverse transcriptase (Applied Biosysytems) using random hexamers. All primers and probes were synthesized by TibMolBiol (Berlin, Germany). Quantification of *BCL11B* mRNA was performed as described elsewhere [24]. Quantification of genes regulated upon *BCL11B* overexpression was performed in Jurkat and huT-78 cell lines. In brief, PCR amplification was performed in a total volume of 25 µl with 2 µl of cDNA, 25 pmol of each primer and SYBR® Green PCR Master Mix. The list of primer sequences is provided in Supplementary Materials and Methods S1. A non-specific signaling resulting from primer-dimer formation was ruled out by analyzing the dissociation curves and agarose gel electrophoresis.

### Analysis of protein levels

Sodium dodecyl sulfate polyacrylamide gel electrophoresis was performed using standard techniques. Equal protein loads were confirmed by Ponceau staining and by immunodetection of ß-actin (Sigma). The Bcl11b protein was detected using antibodies directed against N-terminal, central or C-terminal epitopes (Bethyl Laboratories, TX, USA). The proteins corresponding to the regulated genes confirmed by quantitative RT-PCR were detected with the following antibodies: cleaved Caspase 9 rabbit polyclonal antibody, p57 rabbit polyclonal antibody, p27 rabbit polyclonal antibody, p18 mouse monoclonal antibody, Skp2 rabbit polyclonal antibody, Rb antibody kit, Myc- N rabbit polyclonal antibody (Cell Signaling Technology, MA, USA) and p130 (RBL2) rabbit polyclonal antibody (Santa Cruz Biotechnology, CA, USA). Proteins were visualized by chemiluminescence using rabbit- or mouse-specific Western-*SuperStar*™ Immunodetection system (Applied Biosystems, Life Technologies, CA, USA).

The intracellular staining of Myc-N was performed in fixed and permeabilized cells (Cytofix/Cytoperm, BD Pharmingen, Franklin Lakes, NJ, USA) with N-Myc specific rabbit primary antibody and subsequent immunodetection using goat anti-rabbit APC-labeled antibody (Santa Cruz Biotechnology, CA, USA).

### Sub-cellular localization of full-length BCL11B and BCL11B-derived deletion mutants

The wild-type *BCL11B* coding sequence or alternatively the truncated variants of the gene were cloned in-frame into pEGFP-C1 plasmid vector (Clontech Laboratories, CA, USA) downstream of the enhanced green fluorescent protein (EGFP). The plasmid vectors were transfected into Jurkat cells using the Nucleofector device (Lonza, Switzerland). After 12–24 hours incubation, cells were fixed and the EGFP expression and localization was determined by fluorescence microscopy.

## Supporting Information

Materials and Methods S1 (0.03 MB DOC)
