# Supplementary material for: Increased Expression of Bcl11b Leads to Chemoresistance Accompanied by G1 Accumulation
Source: PLoS One. 2010 Sep 2;5(9):e12532. doi: 10.1371/journal.pone.0012532 (PMC2932720; doi:10.1371/journal.pone.0012532)
Supplement: Materials and Methods S1 — (0.03 MB DOC) [file pone.0012532.s001.doc]

**Supplementary Materials and Methods**

**List of primers used for qRT-PCR assay**

| **NAME** | **SEQUENCE** |
| --- | --- |
| BCL6-F  BCL6-R | ACCTgCAgATggAgCATgTTgT  ATCCggCTgTTgAggAACTCTT |
| CCNG2-F  CCNG2-R | TTTTggACAggTTCTTggCTCT  ggATCACATCATgAgTggATggA |
| CDKN1B-F  CDKN1B-R | AATAAggAAgCgACCTgCAACC  TCTTCTgAggCCAggCTTCTTg |
| CDKN1C-F  CDKN1C-R | gCggCgATCAAgAAgCTgT  TCTTTgggCTCTAAATTggCTCA |
| CDKN2C-F  CDKN2C-R | TgCACAAAATggATTTggAAgg  AgCgAAACCAgTTCggTCTTTC |
| CCNG2-F  CCNG2-R | TTTTggACAggTTCTTggCTCT  ggATCACATCATgAgTggATggA |
| HBP1-F  HBP1-R | TATCgCgACCAgTCCACAAAgT  gAgggCgTgCATAggAATgTAA |
| MYCN-F  MYCN-R | ACCACAAggCCCTCAgTACCTC  gCTTCTCCACAgTgACCACgTC |
| SKP2-F  SKP2-R | gTCCgCAggCCTAAgCTAAATC  AgggAggCACAgACAggAAAAg |
| TP53INP1-F  TP53INP1-R | CATAACTCCTgCCCTggTCTCA  TATgCTgCCCCATTTCATTTTg |
